# Exploring the impact of mindfulness training and a health promotion program on cognitive and mental health of older adults

**DOI:** 10.1002/alz.087687

**Published:** 2025-01-09

**Authors:** Ana Carolina Teixeira Santos, Leandro Gomes, Diana R Pereira, Fabiana Ribeiro, Anabela Silva‐Fernandes, Catarina Godinho, Joana Carvalheiro, Carine Federspiel, Jean‐Paul Steinmetz, Anja K. Leist

**Affiliations:** ^1^ University of Luxembourg, Luxembourg Luxembourg; ^2^ State University of Amazonas, Manaus Brazil; ^3^ University of Minho, Braga Portugal; ^4^ University of Luxembourg, Esch‐sur‐Alzette Luxembourg; ^5^ University of Glasgow, Glasgow United Kingdom; ^6^ Zitha, Luxembourg Luxembourg; ^7^ Zitha, LUXEMBOURG Luxembourg

## Abstract

**Background:**

The positive effects of mindfulness‐based stress reduction (MBSR) on cognition, stress relief, and sleep have been well‐documented. However, there is limited research on its potential benefits for older adults, particularly within vulnerable populations such as migrants. This study aimed to compare the impacts of MBSR with a health promotion program in individuals aged ≥55.

**Method:**

A monocentric, randomized, double‐blind controlled clinical trial was conducted with 89 participants (≥55 years old), all Portuguese‐speaking immigrants in Luxembourg. They were randomly assigned to a 2‐month group intervention, either MBSR (N = 44) or Health Education Program (HEP, n = 45). Data collection occurred at baseline, end of the intervention, and one to three months post‐intervention. Executive functioning was assessed using the Trail Making Test, Stroop color‐word task, and letter‐number sequencing. Secondary outcomes encompassed general cognitive functioning, cortisol level, heart rate variability, and self‐reported affective and mindfulness states.

**Result:**

Of the 125 individuals screened, 89 (mean age: 62.58 years ± 6.08, 63 [70.8%] women) were enrolled, with 75% completing at least one post‐intervention assessment in MBSR and 53% in HEP, included in the modified intention‐to‐treat analysis. The overall dropout rate was 36% and no adverse effects were identified. Time effects were observed in the Letter‐Number Sequencing (*β* = 0.47, SE = 0.23, *t* = 2.09, *p* = 0.04), anxiety (*β* = ‐1.70, SE = 0.49, *t* = ‐3.46, *p* < 0.01), and perceived stress (*β* = ‐2.17, SE = 0.71, *t* = ‐3.05, *p* < 0.01). However, there were no significant group differences or group*time interactions (*p* > 0.05).

**Conclusions:**

Both interventions demonstrated positive impacts on attention, anxiety, and perceived stress among older migrants, indicating that these group interventions have the potential to improve cognitive and affective indicators irrespective of the intervention content. Future studies could further investigate these findings by incorporating an additional control arm with no social contact to help validate the protective effects of group interventions in older vulnerable populations. Our work underscores the significance of positioning these interventions as potential strategies within the realm of dementia prevention.

